# Fossil tabulate corals reveal outcrops of Paleozoic sandstones in the Atlantic Coastal Plain Province, Southeastern USA

**DOI:** 10.1371/journal.pone.0224248

**Published:** 2019-10-24

**Authors:** James E. Landmeyer, Francis Tourneur, Julien Denayer, Mikołaj K. Zapalski

**Affiliations:** 1 Southeast Region, U.S. Geological Survey, Lutz, FL, United States of America; 2 Department of Sciences, University of Liège, Sart-Tilman, Liège, Belgium; 3 Faculty of Geology, University of Warsaw, Zwirki i Wigury, Warszawa, Poland; Southern Illinois University, UNITED STATES

## Abstract

The geologic history of the Southeastern United States of America is missing nearly 350-million-years of rocks, sediments, and fossils. This gap defines the Fall Line nonconformity where Upper Ordovician consolidated rocks are directly overlain by Upper Cretaceous unconsolidated sediments of the Atlantic Coastal Plain Province. Here we begin to fill in the missing geologic record by reporting the discovery of fossils of lower-to-middle Paleozoic tabulate corals (Syringophyllidae) in angular, quartz-rich, ferruginous sandstones that crop out in the Carolina Sandhills Physiographic Province that forms the updip margin of the Atlantic Coastal Plain Province near the Fall Line. These fossils of extinct tabulate corals are the first evidence that Paleozoic (Upper Ordovician–Lower Silurian) sandstones crop out amidst the mostly Mesozoic-to-Cenozoic deposits of the Atlantic Coastal Plain Province of the United States of America. This discovery of Paleozoic fossils and strata in a region in which they were previously entirely unknown offers a more complete insight into the geologic history of the Southern Appalachian Mountains Region, Carolina Sandhills and updip margin of the Atlantic Coastal Plain Province and extends the previously identified range of Syringophyllidae in North America.

## Introduction

**Fig 1:**
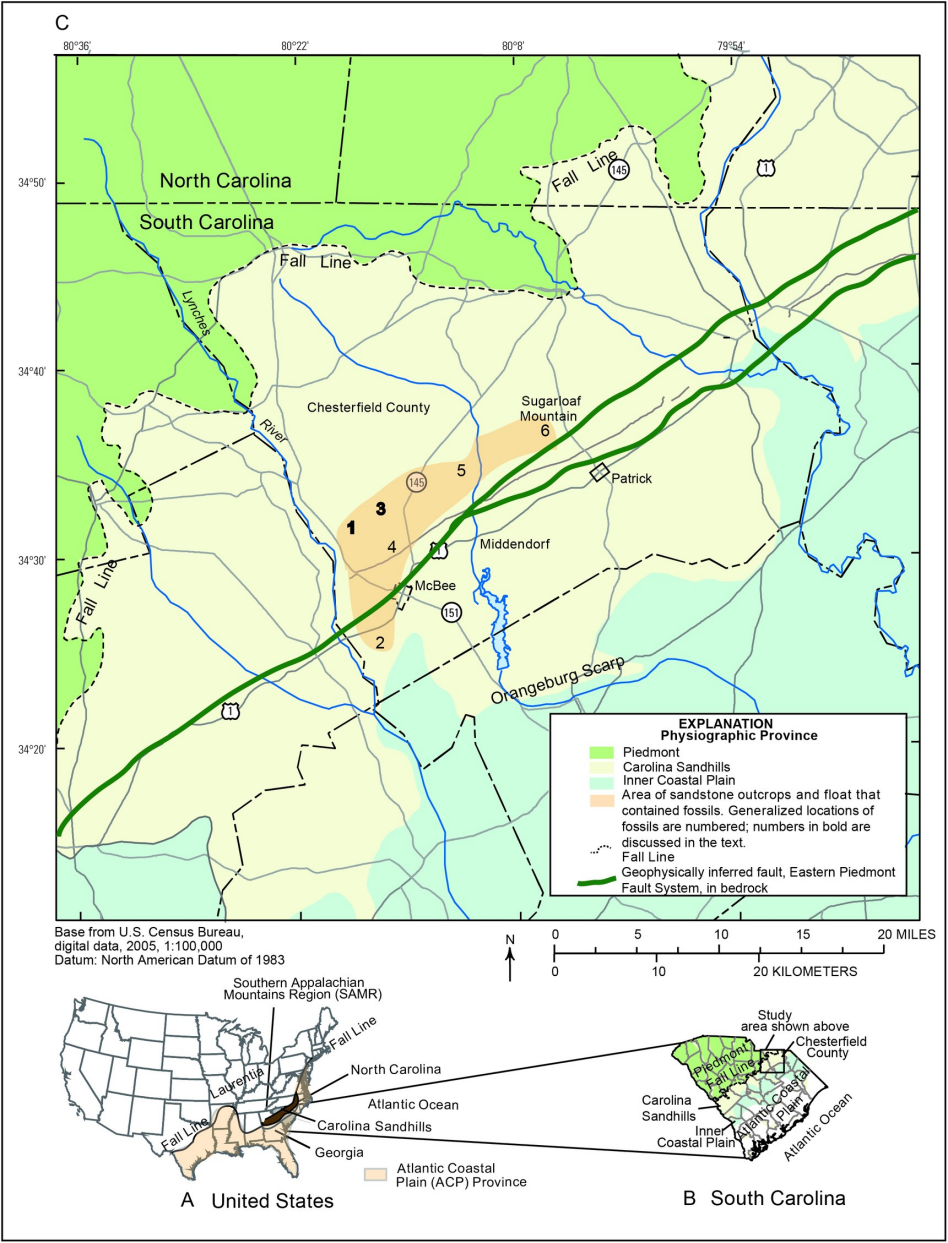
Maps of the study area in the Southeastern United States of America. A. Generalized location of Laurentia, the Southern Appalachian Mountains Region, Atlantic Coastal Plain Province, Carolina Sandhills Physiographic Province, the Fall Line, and Georgia, South and North Carolina, Unites States of America (adapted from [36]). B. Enlarged map of South Carolina that depicts the location of the Carolina Sandhills, Fall Line, Piedmont, Atlantic Coastal Plain, and study area in Chesterfield County. C. Enlargement of study area shown in Fig 1B with generalized locations of the Fall Line, Orangeburg Scarp, geophysically inferred faults of the Eastern Piedmont Fault System, and area of fossiliferous, ferruginous sandstone outcrops with generalized fossil localities labelled 1–6 (localities in bold are discussed in the text).

No fossils from the Paleozoic have been found in situ in the unconsolidated sediments of the Atlantic Coastal Plain (ACP) Province of the United States of America (USA). The oldest fossils from the ACP are found in the Upper Cretaceous and younger sediments at the updip (inland) margin near the Fall Line (Fig 1A), are floral, non-marine, and represent deposition of terrestrial sediments in an upper-delta plain paleoenvironment along a passive margin [1–10]. In contrast, the metaigneous and metasedimentary rocks of the Piedmont Province north of the Fall Line contain lower Paleozoic, and older, fossils. For example, in the Southern Appalachian Mountains Region (SAMR) (Fig 1A), Upper-to-Middle Ordovician and older invertebrate fossils have been found in North Carolina [11–18], Georgia [19, 20], and South Carolina [21–23]. These fossils helped establish temporal constraints of an Upper Ordovician closure of the Iapetus Ocean following accretion of multiple peri-Gondwanan terranes and composite volcanic-arc systems, such as exotic Carolinia, to Neoproterozoic rocks of Laurentia [22, 24–35]. The Fall Line nonconformity, therefore, represents an extensive hiatus in the rock and fossil record from the Lower Silurian to the Lower Cretaceous that has been recognized since at least the 19^th^ century [1–4]. Therefore, it is warranted to report the discovery of fossils from any part of this missing time.

## Geological setting

**Fig 2:**
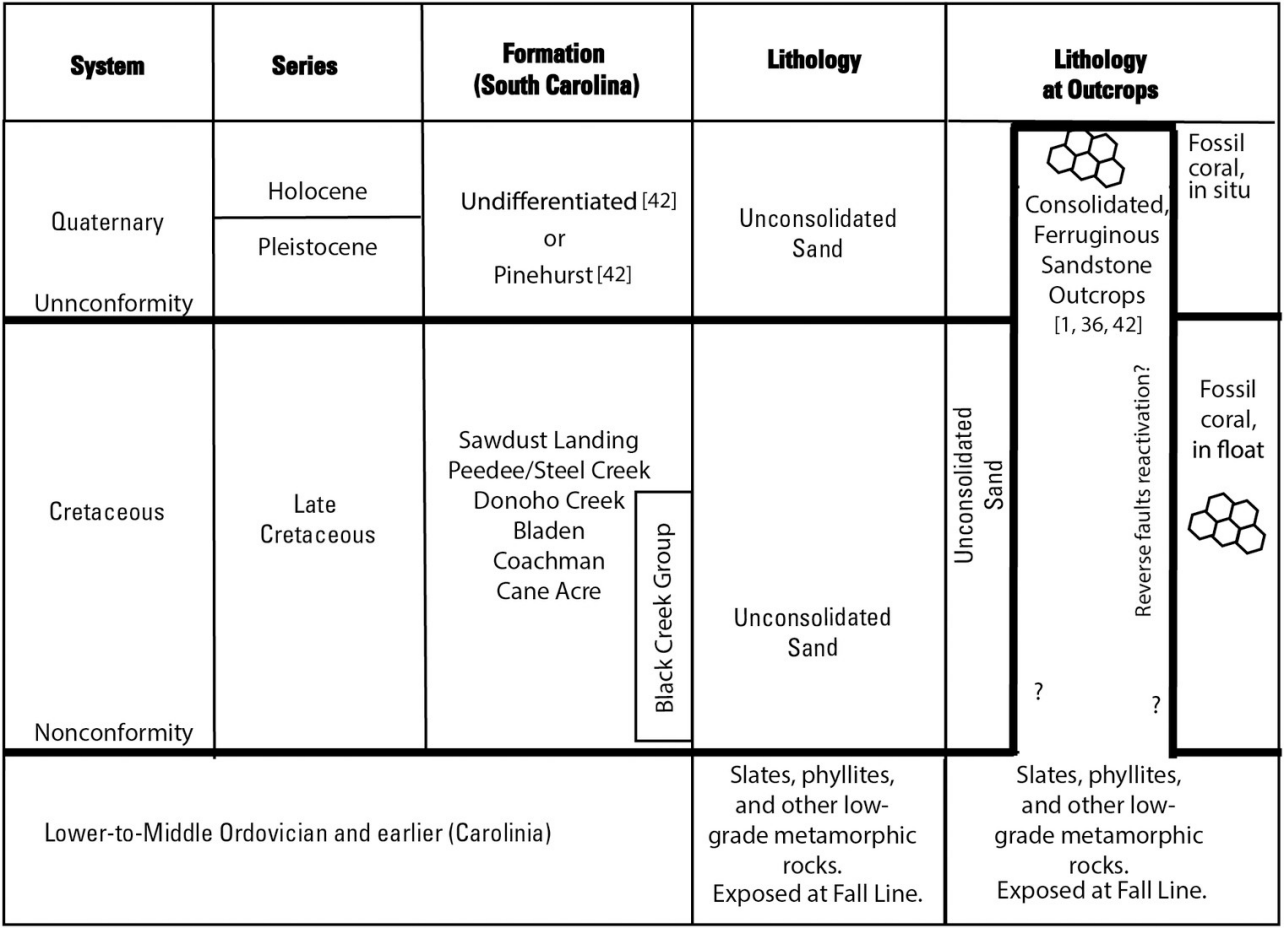
Diagram showing a generalized geologic section of the study area. The formation names are those used in South Carolina [36]. The lithology of the unconsolidated Cretaceous sand is presumably penetrated by the lithologic sequence of the fossiliferous, ferruginous sandstone outcrops expressed at land surface, as proposed in the last column.

The Carolina Sandhills Physiographic Province (Fig 1A) is located in the most updip part of the ACP province adjacent to the Fall Line in Georgia, South Carolina (Fig 1B), and North Carolina and encompasses 22,530 square kilometer (km^2^) and comprises Upper Cretaceous to Quaternary deposits that rest nonconformably on Paleozoic-age rocks of Carolinia, as stated previously [22, 24–31, 33–35] (Fig 2). The thickness of the Upper Cretaceous increases toward the southeast, reaching over 152-m near McBee [37], and extend beneath the sediments of the Inner Coastal Plain at the Orangeburg Scarp. Quaternary-age deposits rest unconformably on the Upper Cretaceous sediments and include the Pinehurst Formation and undifferentiated sediments [36, 38] (Fig 2); the thickness and aerial extent of these sediments ranges from highly variable to absent [39–41]. The lithology of the Upper Cretaceous and Quaternary formations is similar, as the latter is derived from the former. The Upper Cretaceous formations consists of poorly sorted, subangular, medium- to coarse, micaceous (1%), kaolinitic, mature quartz (99%) unconsolidated sand interspersed with lenses of clay and the Quaternary, where present, is considered to represent unconsolidated eolian sand sheets and dunes comprised of reworked Upper Cretaceous material [10, 38, 42].

**Fig 3:**
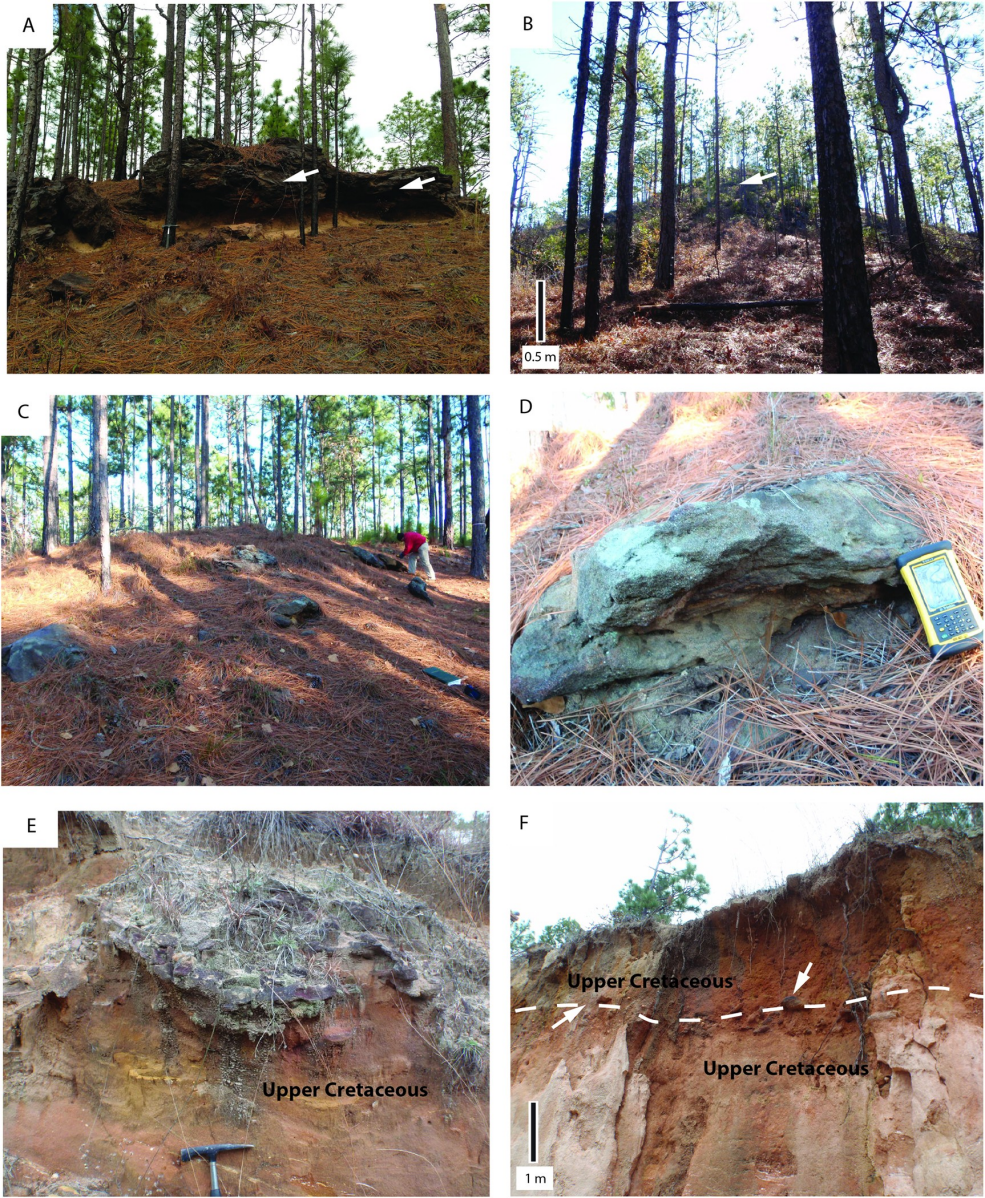
Photographs of ferruginous sandstone outcrops and float. A. Outcrop (labelled no. 1, Fig 1C; arrow points to generalized locations of in situ fossils). Hammer shown for scale. B. Outcrop (labelled no. 6, Fig 1C; arrow points to generalized locations of in situ fossils). C. Float (near outcrop labelled no. 4, Fig 1C). Person shown for scale. D. Float (near outcrop labelled no. 4, Fig 1C). GPS device shown for scale. E. Float (near outcrop labelled no. 2, Fig 1C). Hammer shown for scale. F. Float buried by Upper Cretaceous sediments (near outcrop labelled no. 2; Fig 1C).

The Carolina Sandhills are also characterized by sparsely dispersed outcrops of coarse, iron-cemented (ferruginous) sandstones that create some of the highest altitudes across an otherwise mostly flat region [4] (Fig 3A and 3B). Previous workers [1, 2, 42, 43] consider these enigmatic outcrops to represent either a part of the Upper Cretaceous Cane Acre and Coachman or Quaternary Pinehurst formations, based solely on relative stratigraphic position (Fig 2) with persistence explained by resistance to erosion imparted by the iron cementation. One prominent sandstone outcrop, called Sugarloaf Mountain (location shown on Fig 1C), rises more than 45 meters (m) above the surrounding terrain. The thickness of the sandstone beneath the exposed outcrops is not known (Fig 2), although bedrock is no greater than 61 m below land surface [44] and is often exposed in shallow streambeds near the study area. Ferruginous sandstone float can be found at land surface near the outcrops (Fig 3C and 3D) or below land surface entrained in Upper Cretaceous sediments at lower altitudes (Fig 2, Fig 3E and 3F). Though mapped together with Upper Cretaceous and younger sands by former surveys, it appears that these ferruginous sandstone units are lower-to-middle Paleozoic in age and located above younger formations due to reactivation of reverse faults, as demonstrated here.

## Materials and methods

All in situ fossils and fossiliferous float material were discovered at the peak of or adjacent to ferruginous sandstone outcrops, respectively, near the towns of McBee and Patrick in central South Carolina, USA. The generalized locations of the fossiliferous outcrops are labelled 1‒6 (Fig 1C). The latitude and longitude of each fossil, either discovered in situ or as float, were recorded in the field by using a hand-held global-positioning system (GPS) device and geospatially referenced using a LiDAR coverage. Fossils found in situ were not removed, although fragments were recovered, along with float. The specimens and the locality information are housed at the U.S. Geological Survey (USGS) Caribbean-Florida Water Science Center, 4446 Pet Lane, Suite 108, Lutz, FL, USA. Details of locality information will be made to qualified researchers. No permits were required for the described study, which complied with all relevant regulations. All fossils were discovered by the first author between 2011 and 2018.

Each fossil hand specimen or fragment was examined by using a Leica M205C stereomicroscope (20.5:1 zoom and objective Planapo 0.63x.) illuminated with a Leica LED5000 ring light (80/40). Standard thin sections (30 micrometers (μm), 26 x 46 millimeters (mm)) of representative fossils were prepared (Burnham Petrographics, LLC, Rathdrum, ID). Digital images were made with a Leica M205C integrated 5 Mpixels digital microscope camera (DFC450) and Leica LAS Montage Software. Systematic description and classification follow Hill [45]. The color of iron cementation and morphology of sediment cemented to the fossils are evaluated to understand possible post-burial diagenesis ranging from cementation to low-grade metamorphism.

## Results and discussion

**Fig 4:**
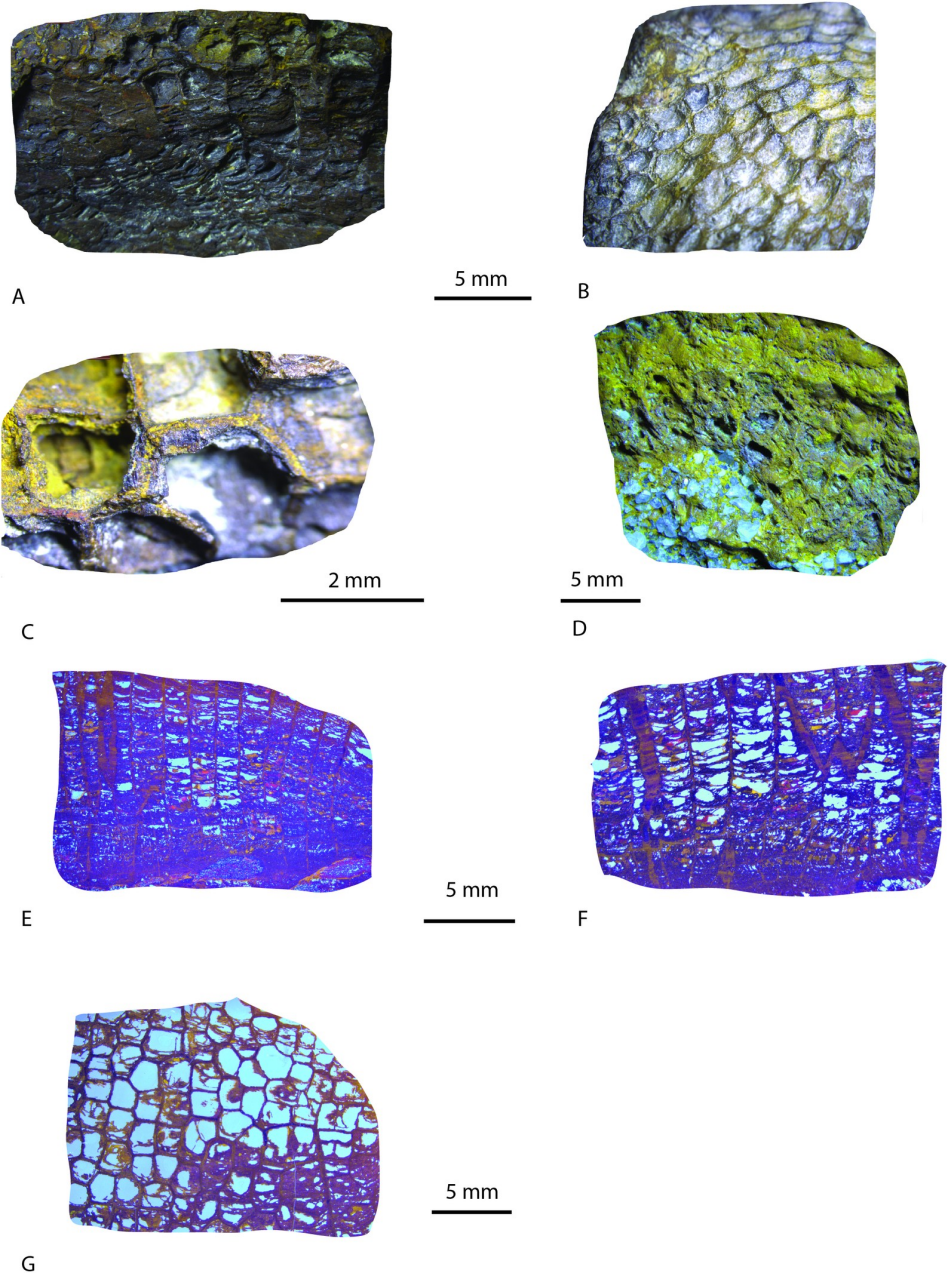
Photographs of tabulate coral fossil hand specimens and thin sections. A. Fragment, massive, hemispherical corallum of polygonal corallites, variable diameters 2 to 3 mm, tabulae numerous, convex, individual corallite budding present, USGSSC-001 (outcrop labelled no. 3, Fig 1C) (×5). B. Fragment, massive corallum, corallites polygonal, variable diameters from 2 to 3 mm, tabulae numerous, convex (funnel-shaped) upward toward calical surface, USGSSC-002 (outcrop labelled no. 3, Fig 1C) (×5). C. Corallum showing separate, unshared calice walls and thecal contact as limonite pseudomorphs of hematite, USGSSC-003 (outcrop labelled no. 3, Fig 1C) (×20). D. Fragment, in situ, massive, corallum of polygonal corallites, variable diameters 2 to 3 mm, large, angular, unsorted quartz grains cemented to material, USGSSC-004 (outcrop labelled no. 1, Fig 1C) (×5). E. Longitudinal section of specimen shown in Fig 4B, corallites, 2 to 3 mm, with complete tabulae. Compares favorably to that shown for *Baikitolites sp*. in Hill [45], Fig 343, 2b, p. F527) (×5). F. Longitudinal section of specimen shown in Fig 4A, corallites, 2 to 3 mm, with complete tabulae. Budding apparent in Fig 4A visible (×5). G. Transverse section of specimen shown in Fig 4A, showing distinct five- to six-sided, “honeycomb-shaped” polygonal calice, closely packed corallites, diameters up to 3 mm. Compares favorably to that shown for *Baikitolites sp*. in Hill [45], Fig 343, 2b, p. F527) (×5). Microstructures were not observed in any specimen.

Specimens in hand (Fig 4A–4D) and thin section (Fig 4E–4G) indicate the fossil corals are characterized by complete tabulae. Although poorly preserved and with no visible septa, the tabulae unequivocally indicate the specimens represent tabulate corals. These skeletal features also demonstrate that the specimens are not hematite nodules or septarian concretions.

The ceroid and polygonal corallites range between 2 and 5 mm (Fig 4A–4G) with horizontal tabulae and no visible septa (Fig 4C and 4G) and strongly resemble corals of the Order Sarcinulida, family Syringophyllidae [45]. Syringophyllidae ranged from the Late Ordovician to Early Silurian [45]. The absence of mural pores (Fig 4C and 4G), presence of semicircular corallites in longitudinal thin section (Fig 4E and 4F), and separation of groups of corallites from neighboring groups by longitudinal lacunae (Fig 4E and 4F) tentatively suggest an attribution to the genus *Baikitolites sp*. [46]; if confirmed, this would be the first record of *Baikitolites sp*. outside of central Asia [47, 48] where association is Late Ordovician. The lack of microstructures precluded a more specific assignment beyond genus; however, the use of microstructures in tabulate systematics is disputable [49, 50]. The systematic paleontology of the fossils is provided herein:

### Systematic paleontology

This is the first occurrence of any tabulate coral in the study area, and the first record of the family Syringophyllidae east of the Appalachian Plateau and Valley and Ridge Provinces.

Class ANTHOZOA [51]

Subclass TABULATA [52]

Order SARCINULIDA

Family SYRINGOPHYLLIDAE [53]

**Genus? *Baikitolites sp***. **[46]**

Fig 4A–4D

#### Material

4 poorly preserved, iron-epigenized specimens as shown (Fig 4A–4D), collected as in situ fragments and float as shown of Fig 3A–3F. Additional specimens not shown were located in situ or as float at Localities 2 and 4–6 (Fig 1C), but the best-preserved specimens are described herein and used to provide thin sections. All fossils found consist of iron (hematite) replacement with partial limonite pseudomorphs after hematite. No organic matter, calcite, or siderite are present which precluded analyses by carbon-isotopic methods; any siderite or glauconite originally present had been mineralized to iron hydroxides. Chemical composition awaits further analyses, although even petrographic evaluation may not reduce uncertainties surrounding post-burial processes as it permits inspection of the last cementation process at the expense of any previous ones. Microfacies present is weak, or is not primary, having been diagenetically changed, almost exclusively, due to transition from goethite to hematite followed by, in some instances, limonite pseudomorphs after hematite (Fig 4C and 4D). Coarse, angular quartz grains were cemented by hematite to the inner and outer part of some of the recovered material (Fig 4D). These quartz grains are larger and more angular than the subangular quartz that comprises the unconsolidated Upper Cretaceous and younger formations in the study area.

#### Material locality

Locality numbers correspond to those shown on Fig 1C. Locality 1 is the peak of a sandstone outcrop exposed at an altitude of about 153 m above the North American Vertical Datum of 1988 (NAVD 88) located northwest of McBee (Fig 1C). Locality 3 is a road cut at an altitude of about 138 m above NAVD 88 along an unimproved public road north of McBee. Additional specimens were located in situ or as float at Localities 2, 4‒6 (Fig 1C). The widespread distribution of the material dispels concerns of a potential non-geologic source of the material, such as non-native fill.

#### Description

Coralla hemispherical (when shape is visible, Fig 4A) or of unknown geometry (in fragments, Fig 4C), cerioid, composed of polygonal corallites, 2–3 mm in diameter. Tabulae numerous, flat or convex, complete and incomplete. Walls even, thin. Septal apparatus not observed. Connecting pores not observed. Microstructures not observed. Budding extrecalicular (Fig 4F).

## Discussion

The material described here seems to be most similar to *Baikitolites sp*., however it differs from both species *B*. *alveolitoides* [46] and *B*. *magnus* [54] by presence of numerous incomplete tabulae. The species described here differs from the latter species by smaller corallite diameters (sometimes up to 5.5 mm, usually smaller [54]). The material presented here is also somewhat similar to *Saffordophyllum* (Tabulata: *Palaeofavositinae*), Late Ordovician in North America [45] from which it differs by absence of pores. The absence of septal apparatus may be caused by diagenesis. The poor preservation of the skeletal elements, epigenetized in iron (hydro)oxides, precludes a more formal identification.

**Fig 5:**
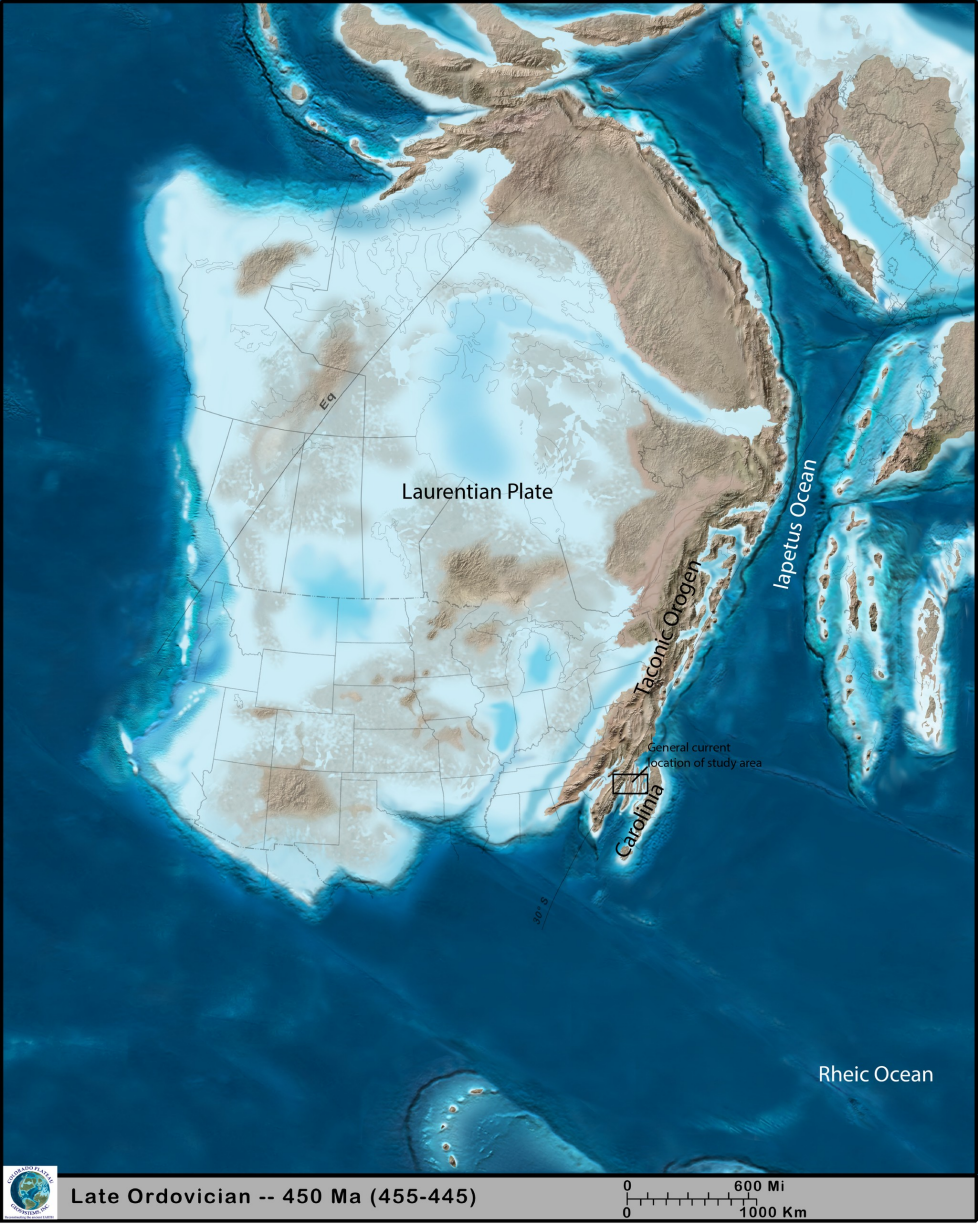
Paleogeographic reconstruction of Laurentia during the Upper Ordovician (450 Ma). Probable location of the study site is shown as square (modified with permission from [59]).

The association of the fossil tabulate coral with coarse, angular sand (Fig 4G) at the outcrops and in float is rare [55, 56] and, therefore, most likely records the depositional event(s) that buried the corals rather than the original growth substrate. The angular sand and lack of large clasts indicate erosion of a proximal granitic highland, such as the southernmost part of the Taconic or Salinic highlands (Fig 5). For example, paleogeographic reconstructions of the Late Ordovician indicate the study area was likely characterized by a narrow, high-energy shelf that would have had significant siliciclastic input from the highlands as sea levels fell [57, 58]. Because delicate skeletal features such as tabulae were preserved, and angular sand grains were found inside and outside of the fossils, indicate the corals were buried rapidly, and locally. Moreover, transport of the fossils, and entombing sand, from distant sources such as the westernmost part of the Allegheny Plateau some 480 kilometers to the northwest would have necessarily obliterated delicate skeletal features and required seaways which are not known to have existed (Fig 5). Moreover, there are no sandstones to the north of the Fall Line. In fact, the rocks to the north of the study site and Fall Line are phyllites of Middle Cambrian to Middle Ordovician age, are not Upper Ordovician to Lower Silurian, and no fossil invertebrates have been found there. Moreover, the current altitude of the Piedmont north of the Fall Line is considerably lower than the study site, as well as most of the ACP.

The source of the iron for fossilization and cementation is subject to speculation because iron supplies to Pre-Cambrian and Paleozoic-age marine sediments has been a matter of considerable debate [60, 61]. For example, assuming the initial source of iron is from the weathering of proximal, ferromag-rich granitic sources, any siderite (FeCO_3_) present at deposition would have been mineralized to goethite and, subsequent to subaerial exposure, hematite. Additional supplies of iron could have been derived from contact metamorphism via pluton emplacement at shallow depths during subsequent orogenies. The transition between diagenesis and metamorphism is gradual and no isothermal boundary can be applied [62]. In any case, it may be possible that other near-shore, biotic or abiotic, components of the Lower Paleozoic exist in the Carolina Sandhills, but are either buried, eroded, or have been mineralized to iron oxides/hydroxides, as has been observed in the Silurian Clinton ironstones [63].

The tectonic feature likely responsible for the uplift of the Paleozoic fossiliferous sandstone could be related to reactivation of the Paleozoic Eastern Piedmont Fault System (EPFS) [64, 65] during the Late Cretaceous-Cenozoic. The EPFS comprises multiple geophysically inferred faults (prominent elongate magnetic anomalies related to ductile deformation during the Paleozoic) beneath most of the ACP sediments in Georgia, South Carolina, and North Carolina, including beneath the study area [65] (Fig 1C). The location of the inferred fault generally coincides with the surface location of the fossiliferous sandstones. The best example of post-Cretaceous reactivation of the EPFS along the Fall Line in South Carolina is the Belair Fault near Augusta, GA. The Belair Fault comprises a series of reverse faults that vertically offset the Upper Cretaceous sediments up to 40 m since the Late Cretaceous [66]. Vertical offsets, most likely from en echelon reverse faulting, as large as 213 m have been reported [67]. In these areas, Piedmont rocks have faulted over ACP strata along high angle reverse faults and is evidenced by brittle deformation [66–68]. In fact, the very linearity of the Fall Line along the length of the ACP has been suggested as evidence of such Cenozoic tectonism [69].

Following post-Cretaceous uplift, the iron-epigenetized corals most likely were then re-covered by fluvial sediments during the Late Cretaceous when sea level was higher than the Silurian [57]. Such deposition would provide for the veneer of sediments that drapes the flanks and bases of most outcrops as described by Sloan [2]. Subsequent re-exposure of most of the outcrops occurred only after sea levels decreased following the Cretaceous and subsequent weathering. Such weathering would also explain the location of fossiliferous float at land surface on the slopes of or near the base of some outcrops (Fig 3C–3E); the float material is composed of the same consolidated, ferruginous sandstone as found at the peaks of the outcrops. Fossiliferous float is also found in a somewhat continuous layer in Upper Cretaceous sediment at depths of 1 to 2 m below land surface (Fig 3F) at short distances from the outcrops and is characterized by the same large, angular, iron-cemented quartz grains that comprise the outcrops.

## Conclusions

This discovery of lower-to-middle Paleozoic tabulate coral fossils in ferruginous sandstones that crop out amidst the much younger Carolina Sandhills of the ACP should provide impetus for the recreation of paleolandmasses and potential linkages to the cause of such rapid burial, perhaps as may be related to the Ordovician-Silurian extinction event, as this initial work documents rapid coral burial as evidenced by fossil association with angular sand grains. The fossils extend the distribution of the Syringophyllidae into North America where they were not previously recognized or recorded. Additional field and laboratory work would facilitate further resolution of the geologic history of the SAMR and ACP in the Southeastern USA to an extent similar to the more fossiliferous strata of Avalonia in the Northern Appalachians.
